# Circulating and dietary advanced glycation end products and obesity in an adult population: A paradox of their detrimental effects in obesity

**DOI:** 10.3389/fendo.2022.966590

**Published:** 2022-12-01

**Authors:** Abduladheem Turki Jalil, Ameer A. Alameri, Rumi Iqbal Doewes, Amr A. El-Sehrawy, Irfan Ahmad, Pushpamala Ramaiah, Mustafa M. Kadhim, Hamzah H. Kzar, R. Sivaraman, Rosario Mireya Romero-Parra, Mohammad Javed Ansari, Yasser Fakri Mustafa

**Affiliations:** ^1^ Medical Laboratories Techniques Department, Al-Mustaqbal University College, Babylon, Hilla, Iraq; ^2^ College of Science, University of Babylon, Babylon, Iraq; ^3^ Faculty of Sport, Universitas Sebelas Maret, Surakarta, Indonesia; ^4^ Department of Internal Medicine, Faculty of Medicine, Mansoura Specialized Medical Hospital, Mansoura University, Mansoura, Egypt; ^5^ Department of Clinical Laboratory Sciences, College of Applied Medical Sciences, King Khalid University, Abha, Saudi Arabia; ^6^ Faculty of Nursing, Umm al- Qura University, Makkah, Saudi Arabia; ^7^ Medical Laboratory Techniques Department, Al-Farahidi University, Baghdad, Iraq; ^8^ Medical Laboratory Techniques Department, Al-Turath University College, Baghdad, Iraq; ^9^ Veterinary Medicine College, Al-Qasim Green University, Al-Qasim, Iraq; ^10^ Department of Mathematics, Dwaraka Doss Goverdhan Doss Vaishnav College, Arumbakkam, University of Madras, Chennai, India; ^11^ Department of General Studies, Universidad Continental, Lima, Peru; ^12^ Department of Pharmaceutics, College of Pharmacy, Prince Sattam Bin Abdulaziz University, Al-kharj, Saudi Arabia; ^13^ Department of Pharmaceutical Chemistry, College of Pharmacy, University of Mosul, Mosul, Iraq

**Keywords:** AGEs, obesity, BMI - body mass index, sRAGE level, cardiovascular disease

## Abstract

**Background:**

The detrimental role of advanced glycation end products (AGEs) against cardio-metabolic health has been revealed in several previous reports. However, the results of studies regarding the association between AGEs and obesity measurements are inconsistent. In the current meta-analysis, we aimed to quantitatively summarize the results of studies that evaluated the association between circulating and dietary AGEs with obesity measurements among the adult population.

**Methods:**

A systematic search from PubMed, Embase, and Scopus electronic databases until 30 October 2022 retrieved a total of 21,429 observational studies. After duplicate removal, title/abstract screening, and full-text reading by two independent researchers, a final number of 18 manuscripts remained to be included in the meta-analysis.

**Results:**

Those in the highest category of circulating AGEs had ~1.5 kg/m^2^ reduced BMI compared with those in the lowest AGEs category [weighted mean difference (WMD): −1.485; CI: −2.459, −0.511; *p* = 0.003], while a nonsignificant increase in BMI was observed in the highest versus lowest category of dietary AGEs (WMD: 0.864, CI: −0.365, 2.094; *p* = 0.186). Also, lower amounts of circulating AGEs in individuals with obesity versus individuals without obesity were observed (WMD: −57.220, CI: −84.290, −30.149; *p* < 0.001). AGE type can be considered as a possible source of heterogeneity.

**Conclusion:**

In the current meta-analysis, we observed an inverse association between circulating AGEs and body mass index among adults. Due to low study numbers, further studies are warranted to better elucidate these results.

## Introduction

Advanced glycation end products (AGEs), also named glycotoxins, are a group of prooxidant, cytotoxic adducts that are involved in the pathogenesis of numerous diseases including diabetes, cardiovascular comorbidities, and obesity (1). AGEs, identified as low-molecular-weight (LMW) and high-molecular-weight (HMW) heterogeneous molecules (2), are formed by non-enzymatic glycation of proteins, amino acids, and nucleic acids; they are formed in high temperatures of foods such as during grilling, roasting, and broiling or frying (3). Numerous studies have revealed the role of AGEs in the development of diabetes complications by raising intracellular reactive oxidative species (ROS), inducing beta cell injury, and malfunction and peripheral insulin resistance (4). Some important types of AGEs include N-ϵ-carboxymethyl lysine (CML), pentosidine, pyrraline, N-ϵ-carboxyethyl lysine (CEL), and methylglyoxal (MGO)-derived hydroimidazolones (MG-H1) (5). AGEs have exogenous or endogenous sources in the body; AGEs formation in the body is through Millard reaction as part of normal metabolism; however, hyperglycemia and oxidative stress could also trigger their formation in the body (6). Dietary AGEs (dAGEs) are important contributors to the body’s AGE pool and are common in modern Western diets containing highly processed foods such as fried and smoked meats, roasted chicken, white French fires potato, broiled lamb, high-sugar and high-fat foods, or foods that are cooked at high temperatures or for long periods (7). Estimation of dietary AGE consumption is possible by measuring one of their main metabolites, carboxymethyl lysine (CML); also, CML’s circulating amounts can be measured with gas chromatography–mass spectrometry (GC-MS) and enzyme-linked immunosorbent assay (ELISA) (8, 9). The association of AGEs with adiposity and obesity risk has been reported in several previous studies; the study by Amin et al. (10), reported a significant positive association between CML and obesity measurements such as body mass index and waist-to-hip ratio. In another study by Uribarri et al. (1), N-carboxymethyl lysine, MGO levels were significantly increased in persons with obesity with more than one other metabolic syndrome criteria but not in obese without metabolic syndrome criteria. Several other studies reported lower CML, as an indicator of circulating AGE concentrations in individuals with obesity (11, 12) or lower BMI in highest versus lowest circulating AGE concentrations (13, 14). In the earliest study that was performed in this field, plasma AGE products were decreased in obese children compared with lean controls (15), whereas Mirmiran et al. (3) and Peterson et al. (8) reported higher BMI in the highest versus lowest circulating AGE concentrations. In one mechanistic study by Gaens et al. (16), it was demonstrated that CML accumulation and the expression of its receptor, RAGE, were higher in the adipose tissue of subjects with obesity compared to those without obesity. The authors concluded that reduced circulating levels of CML observed in subjects with obesity were due to the trapping of CML *via* RAGE in the adipose tissue, resulting in lower circulating CML levels. As mentioned in the above introduction, there are big discrepancies in the results of studies that make it impossible to infer conclusive evidence about the AGE–obesity relationship. Therefore, in the current meta-analysis, we summarized the results of observational studies that evaluated the relationships between circulating and dietary AGEs with obesity measurements among adults.

## Methods and materials

The current report was written according to Preferred Reporting Items for Systematic Reviews and Meta‐Analyses (PRISMA) guidelines (**Supplementary Table 1**) (17). The study protocol’s registration number in the International Prospective Register of Systematic Reviews system (PROSPERO) was CRD42021243323.

### Search strategy and study selection

**Figure 1:**
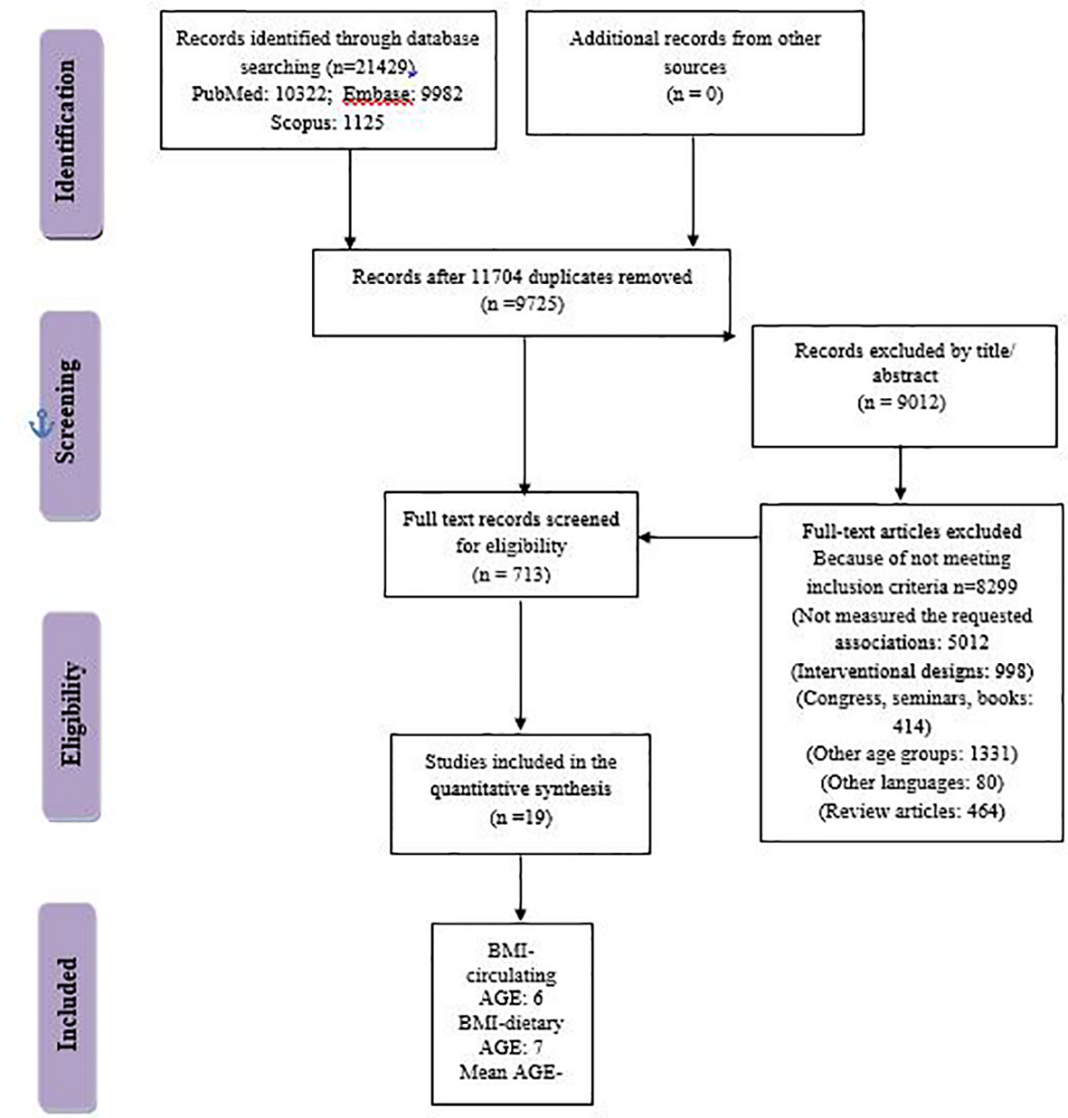
Study flowchart.

A total of 21,429 articles were obtained through a systematic search from PubMed, Embase, and Scopus electronic databases until 30 October 2022. We had no language restrictions. No missing document was found through hand-searching from reference lists of all papers. The search strategy is presented in **Supplementary Table 2**. To avoid missing the studies that measured obesity as a secondary or tertiary measurement variable, in the search strategy, we also included some other keywords related to obesity like waist circumference (WC), waist-to-hip ratio (WHR), abdominal obesity, and central obesity. The retrieved articles were imported into EndNote software. A total of 11,704 articles were removed because of duplication and 9,012 articles were removed according to title/abstract. As a consequence, 713 articles remained to be evaluated by two independent researchers. Finally, 18 manuscripts were included in the final meta-synthesis (**Figure 1**).

### Inclusion and exclusion criteria

In the current systematic review and meta-analysis, included studies were observational studies with a cross-sectional design; evaluated the relationship between circulating or dietary AGEs and obesity measurements like body mass index (BMI), fat mass, waist circumference (WC), or waist-to-hip ratio (WHR); and were conducted among adults. The included studies also provided the mean ± standard deviation (SD) of BMI, fat mass, WC, or WHR of those in the lowest versus highest categories of circulating or dietary AGE and/or provided the mean ± SD of AGE in adults with or without obesity.

The studies with interventional design, case reports, and case series, experimental and *in vitro* studies, reviews, letters to editors, abstracts of congress or seminars, and short communications were excluded. The PICO (patients, intervention, control-comparator, and outcome) model for the studies’ selection is presented in **Table 1**.

**Table 1:** The PICO criteria used for the systematic review.

PICO criteria	Description
Participants	Adult population
Exposure (Interventions)	Highest category of circulating or dietary AGE
Comparisons	Lowest category of circulating or dietary AGE
Outcome	BMI
Study design	Observational studies with the design of cross-sectional, case–control, or cohort

AGE, advanced glycation end products; BMI, body mass index.

### Data extraction and quality assessment of included studies

The retrieved articles were extracted by three independent researchers using a standard Excel extraction datasheet. Some of the characteristics of retrieved articles include first author name, year of publication, journal name, country, age of participants, study design, the total number of participants, adjusted covariates, gender, study setting, and circulating or dietary AGE measurement tools; the main findings of the studies were extracted. The methodological quality of the included studies was assessed using the Agency for Healthcare Research and Quality (AHRQ) checklist (18) (**Table 2**).

**Table 2:** Agency for Healthcare Research and Quality (AHRQ) checklist to assess quality of the cross-sectional studies.

ARHQ Methodology Checklist items for Cross-Sectional study	Huang QF (19)	Semba RD (20)	Semba RD (21)	Semba RD (22)	Moy KA (23)	Amin MN (10)	Davis KE (24)	HanssenNM (25)	Luft VC (11)	Mahmoudinezhad M (14)	Foroumandi E (12)	Cordova R (13)	Mirmiran P (3)	Peterson LL (8)	Scheijen J (9)
1) Define the source of information (survey, record review)	⊕	⊕	⊕	⊕	⊕	⊕	⊕	⊕	⊕	⊕	⊕	⊕	⊕	⊕	⊕
2) List inclusion and exclusion criteria for exposed and unexposed subjects (cases and controls) or refer to previous publications	⊕	⊕	⊕	⊕	⊕	⊕	⊕	⊕	⊕	⊕	⊕	⊕	⊕	⊕	⊕
3) Indicate time period used for identifying patients	⊕	⊕	⊕	⊕	⊕	⊕	⊕	⊕	⊕	⊕	⊕	⊕	⊕	⊕	⊕
4) Indicate whether or not subjects were consecutive if not population-based	U	⊕	U	U	⊕	U	⊕	U	⊕	⊕	⊕	⊕	⊕	⊕	⊕
5) Indicate if evaluators of subjective components of study were masked to other aspects of the status of the participants	U	U	U	U	U	U	U	U	U	U	U	U	U	U	U
6) Describe any assessments undertaken for quality assurance purposes (e.g., test/retest of primary outcome measurements)	**U**	U	**U**	**U**	**U**	U	U	U	**U**	U	U	U	U	U	U
7) Explain any patient exclusions from analysis	⊕	U	⊕	⊕	⊕	⊕	⊕	⊕	⊕	⊕	⊕	⊕	⊕	⊕	⊕
8) Describe how confounding was assessed and/or controlled.	⊕	⊕	⊕	⊕	⊕	U	U	⊕	⊕	⊕	⊕	⊕	⊕	⊕	⊕
9) If applicable, explain how missing data were handled in the analysis	U	U	U	⊕	⊕	U	U	⊕	⊕	⊕	⊕	⊕	⊕	⊕	⊕
10) Summarize patient response rates and completeness of data collection	⊕	U	⊕	⊕	⊕	⊕	⊕	⊕	⊕	⊕	⊕	⊕	⊕	U	U
11) Clarify what follow-up, if any, was expected and the percentage of patients for which incomplete data or follow-up was obtained	–	–	–	–	–	–	–	–	⊕	–	–	–	–	–	–
**Total score**	**6**	**5**	**6**	**8**	**8**	**5**	**6**	**8**	**9**	**8**	**8**	**8**	**8**	**7**	**7**

U, unclear; ⊕, Yes.

### Statistical analysis

Data analysis was performed by STATA version 13 (STATA Corp, College Station, TX, USA). *p*-values less than 0.05 were considered statistically significant. The mean and SDs of the variables were used to calculate the unstandardized effect size calculated by weighted mean difference (WMD) with a 95% confidence interval (CI). When, the median and range were reported instead of mean and SD, the method of Hozo et al. (26) was used for mean and SD estimation. Walter and Yao’s method was also used for calculating missing SDs as an improved “range” method (27, 28). An equal number of participants in each category was assumed if the number of participants in categories was not provided. Cochran’s *Q* and *I* ^2^ tests were used considering the following heterogeneity measurements: no heterogeneity for *I* ^2^ < 25%, moderate heterogeneity for *I* ^2^ = 25%–50%, and large heterogeneity for *I* ^2^ > 50% (29). For significant heterogeneities of either the *Q* statistic with *p* < 0.1 or *I* ^2^ > 50%, the random-effects model was used (30). Subgrouping approaches were also performed to identify the source of heterogeneity. Begg’s Funnel plots followed by Begg’s adjusted rank correlation and Egger’s regression asymmetry tests were used for the assessment of publication bias.

## Results

### Study characteristics

In the two-class meta-analysis of the comparison of BMI between the highest versus lowest circulating AGE categories, six studies with 10,615 total participants were included (19–23, 25). The general characteristics of included studies are presented in **Table 3**. Only in the study by Huang et al. (19) was BMI higher in the highest versus lowest circulating AGE categories. In the other five studies, those with higher circulating AGE concentrations had significantly lower BMI. The main AGE that was measured in most of the studies was CML, which was measured by enzyme-linked immunosorbent assay (ELISA). In the two-class meta-analysis of the comparison of BMI between the highest versus lowest dietary AGE values, seven studies with 343,595 individuals were included. The study by Cordova et al. (13), reported the comparison of BMI between categories of three different types of dietary AGE, namely, CML, CEL, and pentosidine. Therefore, it was included as three independent studies. Most of the studies reported higher BMI in the highest versus lowest categories of dietary AGE values (**Table 3**). In the two-class meta-analysis of the comparison of mean circulating AGE in individuals with or without obesity, five individual studies were included with 895 participants. In the study by Amin et al. (10), CML was compared in both obese diabetic patients and obese nondiabetic patients with their corresponding controls. However, we excluded diabetes and just the results of the comparison of CML between obese versus non-obese individuals were reported. Therefore, their study was included as two independent studies, and accordingly, significantly higher CML was observed in diabetic patients with obesity compared with others. Similarly, the study by Foroumandi et al. (12) measured two different types of AGEs (e.g., CML and pentosidine); they observed significantly lower CML and pentosidine in individuals with overweight/obesity versus individuals without overweight/obesity.

**Table 3:** Characteristics of studies included in the current systematic review.

Studies that reported the comparison of BMI between highest versus lowest circulating AGE categories									
First Author/Year	Country	Journal	Study Population	Gender	Age	AGE assessment methods	Number	AGE type	Main finding
Hanssen, NM/2015 (25)	Denmark	*Diabetes*	Healthy	Both	21–64	ELISA	536	CML, CEL, pentosidine	Significantly lower BMI in highest versus lowest AGE tertiles (*p* < 0.001)
Huang QF/2016 (19)	China	*J Hum Hypertens*	Healthy	Both	53–56	ELISA	1,051	CML, CEL, pentosidine	Significantly higher BMI in highest versus lowest AGE concentrations (*p* = 0.03)
Moy KA/2013 (23)	Finland	*Hepatology*	Healthy	Both	50–69	ELISA	485	CML	Significantly lower BMI in highest versus lowest CML-AGE tertiles (*p* = 0.004)
Semba RD/2011 (20)	USA	*J Nutr*	Healthy	Both	26–93	ELISA	592	CML	Significantly lower BMI in highest versus lowest CML-AGE tertiles (*p* = 0.0002)
Semba RD/2014 (21)	USA	*J Nutr*	Healthy	Both	≥66	ELISA	4,907	CML	Significantly lower BMI in highest versus lowest AGE tertiles (*p* < 0.001)
Semba RD/2016 (22)	USA	*JAMA Ophthalmol*	Healthy	Both	70–79	ELISA	3,044	CML	Significantly lower BMI in highest versus lowest CML tertiles (*p* < 0.0001)
***Studies that reported the comparison of BMI between highest versus lowest dietary energy-adjusted AGE categories***									
Cordova R/2020 (13)	10 European countries	*Eur J of Nutr*	Healthy	Both	25–70	Quantitative DQ, semi-quantitative FFQ and 7–14 day FR	255,170	CEL, CML, and MG-H1	Higher BMI in highest versus lowest quintile of dietary CEL while lower BMI in highest versus lowest CML and MG-H1 quintiles
Mahmoudinezhad M/2021 (14)	Iran	*Sci Rep*	Healthy	Both	18–50	Semi-quantitative FFQ	347	CEL, CML, and MG-H1	Non-significantly lower BMI in highest versus lowest dietary AGE tertiles (*p* = 0.47)
Mirmiran P/2019 (3)	Iran	*MJIRI*	Healthy	Both	19–70	Semi-quantitative FFQ	4,080	CEL, CML, and MG-H1	Significantly higher BMI in highest versus lowest dietary AGE quartiles (*p* = 0.032)
Peterson LL/2020 (8)	USA	*Cancer*	Healthy	Women	50–71	Semi-quantitative FFQ	183,548	CEL, CML, and MG-H1	Higher BMI in highest versus lowest quintile of dietary AGE
Scheijen J/2018 (9)	Netherlands	*Clin Nutr*	Healthy	Both	18–94	Semi-quantitative FFQ	450	CEL, CML, and MG-H1	No significant difference between highest versus lowest dietary AGE tertiles (*p* = 0.690)
***Studies that reported the comparison of mean AGEs between obese and non-obese individuals***									
Amin MN/2011 (10)	Egypt	*Int J Biomed Sci*	Obese	Both	46–48	ELISA	30	CML	Significantly higher CML in obese versus non-obese individuals (*p* < 0.05)
Amin MN/2011 (10)	Egypt	*Int J Biomed Sci*	Obese diabetic	Both	46–48	ELISA	58	CML	Significantly higher CML in diabetic-obese versus diabetic non-obese individuals (*p* < 0.05)
Davis KE/2014 (24)	USA	*Nutr Res*	Healthy	Both	18–45	ELISA	77	CML	Non-significantly higher CML in overweight and obese versus normal weight individuals
Luft VC/2016 (11)	USA	*Diab Med*	Healthy	Both	45–64	ELISA	1,057	CML	Non-significantly lower CML in overweight and obese versus normal weight individuals
Foroumandi E/2019 (12)	Iran	*PLoS One*	Healthy	Both	≥20	ELISA	90	CML	Significantly lower CML in overweight/obese versus non overweight/obese individuals (*p* < 0.05)
Foroumandi E/2019 (12)	Iran	*PLoS One*	Healthy	Both	≥20	ELISA	90	Pentosidine	Significantly lower pentosidine in overweight/obese versus non overweight/obese individuals (*p* < 0.05)

sRAGE, soluble receptor for advanced glycation end products; esRAGE, endogenous secretory receptor for advanced glycation end products; BMI, body mass index; ELISA, enzyme-linked immunosorbent assay; CML, N-ϵ-carboxymethyl lysine; CEL, N-ϵ-carboxyethyl lysine.

*All of the included studies had a cross-sectional design.

### Results of meta-analysis

**Figure 2:**
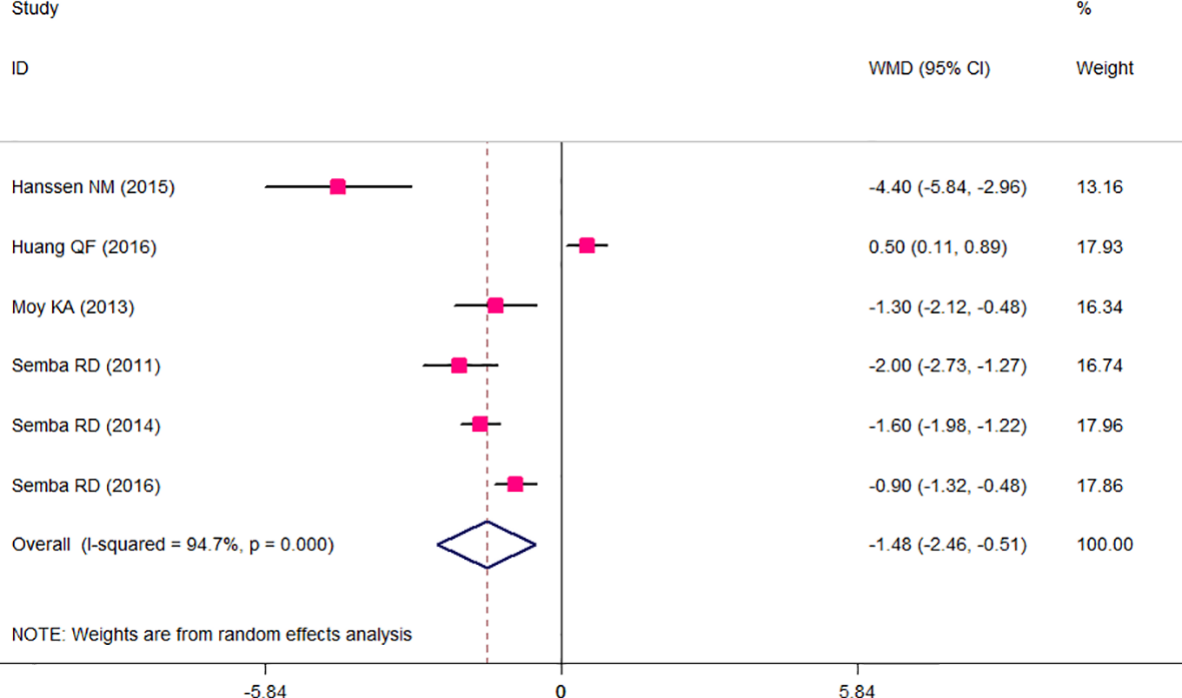
Weighted mean difference (WMD) with 95% confidence interval (CI) of body mass index (BMI) in highest versus lowest categories of circulating advanced glycation end products (AGEs). *I* ^2^ represents the degree of heterogeneity.

**Figure 3:**
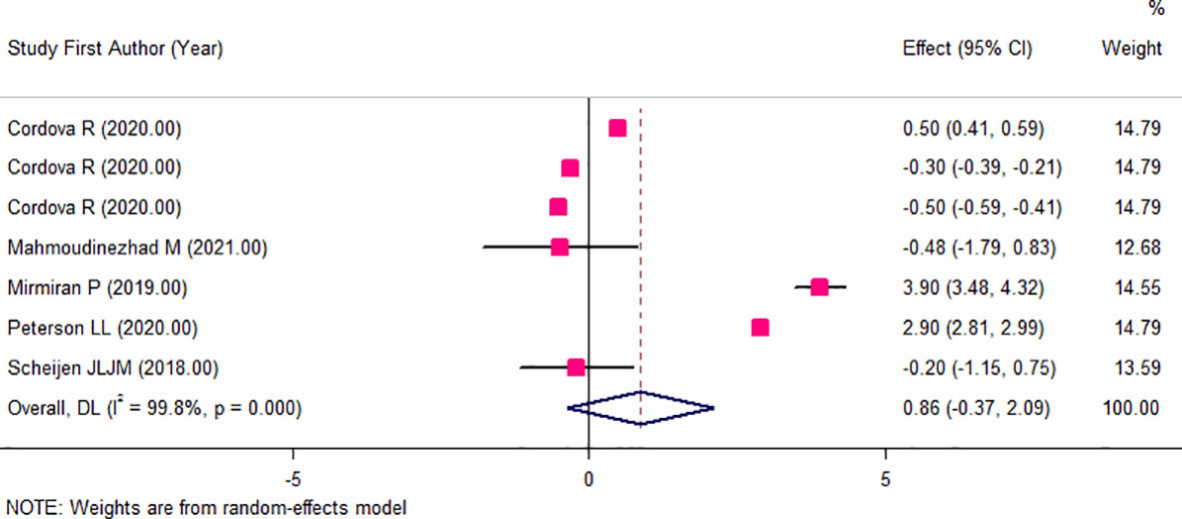
Weighted mean difference (WMD) with 95% confidence interval (CI) of body mass index (BMI) in highest versus lowest categories of dietary advanced glycation end products (AGEs). *I* ^2^ represents the degree of heterogeneity.

**Figure 4:**
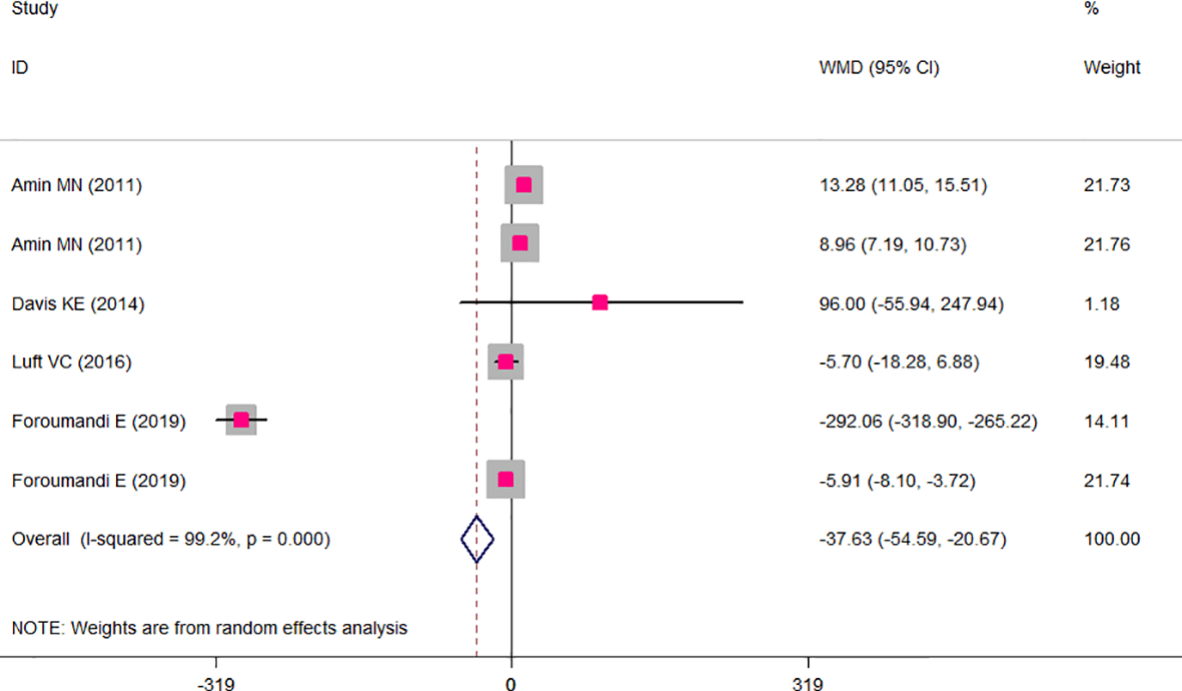
Weighted mean difference (WMD) with 95% confidence interval (CI) of mean circulating advanced glycation end products (AGEs) in obese versus non-obese individuals. *I* ^2^ represents the degree of heterogeneity.

The results of the two-class meta-analysis are presented in **Figures 2**–**4**. As shown in **Figure 2**, being in the highest category of circulating AGEs was accompanied by ~1.5 kg/m^2^ reduced BMI in apparently healthy adults (WMD: −1.485; CI: −2.459, −0.511; *p* = 0.003). However, in the comparison of BMI in different categories of dietary AGEs (**Figure 3**), the result was a nonsignificant increased BMI in the highest versus lowest category of dietary AGEs (WMD: 0.864, CI: −0.365, 2.094; *p* = 0.186). The comparison of circulating AGEs in individuals with or without obesity, and lower amounts of circulating AGEs in individuals with obesity versus those without obesity were reported (WMD: −57.220, CI: −84.290, −30.149; *p* < 0.001; **Figure 4**). Because of the high heterogeneity values in these meta-analyses, we performed a subgroup analysis, and the results are shown in **Tables 4**–**6**. AGE type can be considered as a possible source of heterogeneity in the comparison of BMI in the highest versus lowest AGE categories. However, other parameters could not reduce heterogeneity in a meaningful way and, therefore, possibly are not heterogeneity sources.

**Table 4:** Subgroup analysis for the comparison of BMI between highest versus lowest category of circulating AGE.

Group	No. of studies^*^	WMD (95% CI)	*p*_within group_	*p*_between group*_	*p*_heterogeneity_	*I* ^2^, %
**Total**	6	−1.485 (−2.459, −0.511)	0.003		< 0.001	98.2
**Continent**	** **			< 0.001		
Europe	2	−2.791 (−5.827, 0.245)	0.072		< 0.001	92.6
USA	3	−1.453 (−2.052, −0.854)	< 0.001		0.01	78.1
Asia	1	0.500 (0.107, 0.893)	0.013		–	–
**Baseline BMI (kg/m^2^)**	** **	** **	** **	< 0.001	** **	** **
≤26	3	−0.910 (−2.595, 0.775)	0.29		< 0.001	95.3
>26	3	−2.004 (−3.111, −0.896)	< 0.001		< 0.001	91.3
**AGE type**				< 0.001		
CML	4	−1.416 (−1.893, −0.940)	< 0.001		0.027	67.2
CML, CEL, Pentosidine	2	−1.899 (−6.700, 2.902)	0.438		< 0.001	97.6
**Age group**				< 0.001		
≥50	3	−1.267 (−1.764, −0.771)	< 0.001		0.053	66
≥20	3	−1.886 (−4.394, 0.622)	0.141		< 0.001	97.2
**Sample size**				< 0.001		
<1,000	3	−2.433 (−3.848, −1.018)	0.001		0.001	85.2
≥1,000	3	−0.667 (−1.902, 0.568)	0.29		< 0.001	96.5
**Study quality**				< 0.001		
Moderate	3	−1.018 (−2.613, 0.577)	0.211		< 0.001	97.1
High	3	−2.034 (−3.558, −0.511)	0.009		< 0.001	90.5

*All of the included studies had a cross-sectional design.

**Table 5:** Subgroup analysis for the comparison of mean circulating AGE between obese and non-obese participants.

Group	No. of studies^*^	WMD (95% CI)	*p*_within group_	*p*_between group*_	*p*_heterogeneity_	*I* ^2^, %
**Total**	5	−57.220 (−84.290, −30.149)	< 0.001		< 0.001	99.3
**Continent**	** **			< 0.001		
Egypt	1	11.064 (6.832, 15.296)	< 0.001		0.003	88.7
USA	2	15.808 (−65.590, 97.205)	0.703		0.191	41.5
Iran	2	−148.66 (−429.08, 131.76)	0.299		< 0.001	99.8
**Baseline AGE (ng/ml)**	** **	** **	** **	< 0.001	** **	** **
<100	2	5.448 (−5.334, 16.229)	0.322		< 0.001	98.8
≥100	3	−74.36 (−305.80, 157.07)	0.529		< 0.001	99.4
**AGE type**				< 0.001		
CML	4	−50.59 (−73.390, −27.792)	< 0.001		< 0.001	99.2
Pentosidine	1	−5.910 (−8.104, −3.716)	< 0.001		–	–
**Age group**				< 0.001		
<50	2	11.133 (6.852, 15.413)	< 0.001		0.007	80.1
≥50	1	−5.700 (−18.275, 6.875)	0.374		–	–
Both	2	−148.66 (−429.08, 131.76)	0.299		< 0.001	99.8
**Sample size**				< 0.001		
<80	2	11.133 (6.852, 15.413)	< 0.001		0.007	80.1
≥80	3	−99.84 (−198.448, −1.232)	0.047		< 0.001	99.5
**Study quality**				< 0.001		
Moderate	2	11.133 (6.852, 15.413)	< 0.001		0.007	80.1
High	3	−99.84 (−198.448, −1.232)	0.047		< 0.001	99.5

*All of the included studies had a cross-sectional design.

**Table 6:** Subgroup analysis for the comparison of BMI between highest versus lowest dietary AGE.

Group	No. of studies^*^	WMD (95% CI)	*p*_within group_	*p*_between group*_	*p*_heterogeneity_	*I* ^2^, %
**Total**	7	0.864 (−0.365, 2.094)	0.168		<0.001	99.8
**Continent**	** **			<0.001		
Europe	4	−0.115 (−0.663, 0.432)	0.68		<0.001	98.8
Asia	2	1.756 (−2.536, 6.047)	0.423		<0.001	97.4
USA	1	2.900 (2.813, 2.987)	<0.001		–	–
**Baseline BMI (kg/m^2^)**	** **	** **	** **	<0.001	** **	** **
<28	3	−0.100 (−0.698, 0.498)	0.743		<0.001	99.2
≥28	4	1.696 (0.402, 2.989)	0.01		<0.001	96.6
**Age group**				<0.001		
20–70	5	0.677 (−0.057, 1.411)	0.07		<0.001	99.3
40–70	2	1.388 (−1.649, 4.425)	0.37		<0.001	97.5
**Sample size**				<0.001		
<1,000	2	1.276 (−2.034, 4.585)	0.45		<0.001	96.1
≥1000	5	0.686 (−0.035, 1.408)	0.062		<0.001	99.3
**Study quality**				<0.001		
Moderate	2	1.388 (−1.649, 4.425)	0.37		<0.001	97.5
High	5	0.677 (−0.057, 1.411)	0.07		<0.001	99.3

*All of the included studies had a cross-sectional design.

## Discussion

**Figure 5:**
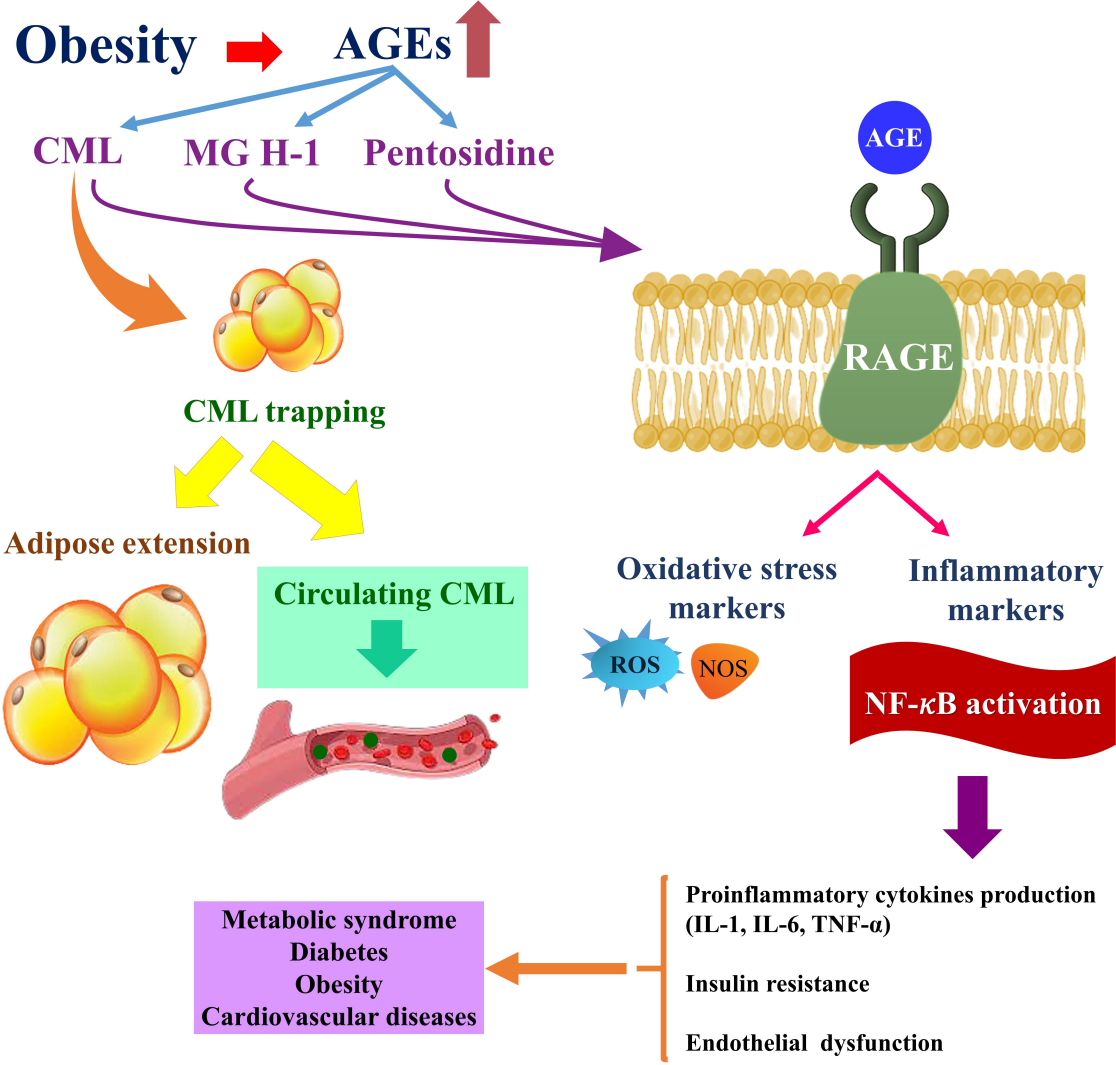
Obesity-related complications are exacerbated by the accumulation of AGEs in adipose tissue. Obesity-related CML accumulation and CML-RAGE-mediated activation of intracellular signaling pathways in adipocytes may promote inflammatory signaling in adipose tissue. The dysregulation of pro-inflammatory and anti-inflammatory cytokines that results may then contribute to the development of obesity-related complications such as metabolic syndrome, diabetes, and cardiovascular diseases (42). On the other hand, trapping CML in adipose tissue reduces serum levels of CML in the bloodstream and expands adipose tissue. Because of the negative relationship of fat mass with serum CML concentrations, serum CML levels may be lower among the obese compared with those who are lean despite a higher intake of AGE-rich foods (20). AGE, advanced glycation end product; CML, carboxymethyl-lysine; MG H1, methylglyoxal-derived hydroimidazolone; RAGE, receptor for advanced glycation end products; NF-κB, nuclear factor κB; ROS, reactive oxygen species; NOS, nitric oxide; IL, interleukin; TNF-α, tumor necrosis factor-alpha.

In the current meta-analysis, we observed an inverse association between BMI and circulating AGE concentrations in a healthy adult population in two meta-analyses with 11,510 participants. At first glance, these findings seem to be in contrast with the pre-established detrimental effects of AGE in promoting obesity and its related disorders; in the previous meta-analysis of interventional studies by Sohouli et al. (31), low-AGE diets significantly reduced weight and BMI in subjects with obesity, polycystic ovary syndrome, or diabetes. They did not evaluate circulating AGEs; however, we observed a nonsignificant higher BMI in the highest versus lowest dietary AGEs categories. Numerous previous studies reported higher BMI in those with high dietary AGE consumption (3, 8, 13). High dietary AGE content promotes chronic inflammation and insulin resistance in subjects with obesity and helps the transition from healthy obesity to unhealthy obesity with susceptibility to metabolic syndrome; in other words, the inverse association between circulating AGEs and obesity is the concept of healthy versus non-healthy or metabolic syndrome-prone obesity and circulating AGEs that was first proposed by Uribarri et al. (1); they observed significantly higher circulating AGEs (CML and MG-H1) only in subjects with obesity with at least one component of metabolic syndrome versus normal weight individuals, whereas no significant difference was observed among AGE concentrations of individuals with obesity without any metabolic syndrome components versus normal weight individuals. The authors suggested that in individuals with obesity with one metabolic syndrome component, there were also higher circulating amounts of inflammatory factors including leptin, tumor necrosis factor (TNF)-α, and receptor for the advanced glycation end product (RAGE) that promotes deteriorating effects of AGE in metabolic syndrome-prone patients with obesity. AGEs are potent inducers of inflammation and oxidative stress, and this is mostly done *via* the activation of the AGE/RAGE axis, excessive stimulation of the PI3K-PKB-IKK pathway, and NF-κB binding on the RAGE promoter (32). Also, higher CML concentrations and RAGE expression in the adipose tissue of subjects with obesity lead to the assumption of trapping of CML-AGE *via* RAGE in the adipose tissue, which causes reduced circulating levels of CML in subjects with obesity (16). However, in the experimental model conducted by Tessier et al. (33), mice were exposed to chronic oral CML administration and showed an accumulation of CML in all tissues except fat. The rate of deposition was high in the kidneys, intestine, and lungs, and low (<5 μg/g) in the heart, muscle, and liver, and they reported that this accumulation was not RAGE dependent. It was an animal model and not the usual dietary intake of AGEs; therefore, the results might not be distributed to human models and further studies should be performed. It is mostly known as “dicarbonyl stress in obesity”, which suggests that the increased MGO formation from glyceroneogenesis on adipose tissue and liver and the decreased glyoxalase-1 activity in obesity possibly cause dicarbonyl stress in white adipose tissue with increased dicarbonyl proteome and, as a consequence, increased transcapillary escape rate of albumin and total body interstitial fluid volume in obesity; this phenomenon will make the levels of glycation of plasma protein (e.g., AGEs) become unreliable indicators of glycation status in obesity (34). Also, another important issue is the type of AGEs that are measured in different studies. As shown in our study, most of the studies that reported an inverse association between circulating AGEs and BMI measured CML as the main AGEs. However, it is well-known that serum CML concentration is strongly affected by body fat; actually, CML is preferentially deposited in fat tissue and adipocytes affect the metabolism of AGE (31). In this situation, circulating CML does not reflect the total body content of CML, and the most important AGE that will be a more reliable indicator of the body’s AGE status is MG-H1, which, in most of the studies, is only measured in the dietary assessment of AGE and not its circulating amounts (35). MGO leads mainly to the formation of MGO-derived cyclic hydroimidazolones (MG-Hs) in three well-known isoforms of MG-H1, MG-H2, and MG-H3. MG-H1, the most abundant and important MGO-derived AGE, accounts for more than 90% of all MGO adducts (36, 37). Both free MG-Hs and MG-H-modified proteins serve as ligands for the receptor of AGEs (RAGE) (38). MG-H1 is strongly associated with increased oxidative stress and even very small increases of MG-H1 modifications of mitochondrial proteins have been linked to a two- to threefold increase in oxidative stress that has a great role in the pathogenesis of chronic diseases like diabetes, chronic kidney disease, and atherosclerosis (39, 40). MG-H1 increases the generation of superoxide anion radicals and serves as an early indicator of the progression of diabetic nephropathy lesions and glomerular basement membrane increase (41). A summary of these mechanistic pathways is illustrated in **Figure 5**. In the current meta-analysis, all of the included studies had moderate or high study quality and no study had poor quality. In the subgrouping, we demonstrated the possible role of the AGE type as a source of heterogeneity. However, because of the low number of studies in each subgroup, making a reliable conclusion will be limited. In conclusion, in the current systematic review and meta-analysis, for the first time, we summarized the studies that evaluated the association between obesity with circulating and dietary AGE values, and we found a negative association between BMI and circulating AGE concentrations among adults. Further observational studies are warranted to make this conclusion more reliable. Also, it is suggested that, in other studies, MG hydroimidazolone-1 must be measured instead of CML as the main AGE to better elucidate the body’s AGE status.

## Data availability statement

The raw data supporting the conclusions of this article will be made available by the authors, without undue reservation.

## Author contributions

ATJ and AAE supervised the project and were involved in hypothesis generation, searching and conceptualization. AAA and RID were involved in extraction and data analysis. IA, PR, MMK and HHK were all involved in data analysis, extraction and searching through different search engines, RMRP and MJA were involved in manuscript writing and edition. YFM was involved in manuscript revision and English edition. All of the authors have read and approved the final draft of the article to be published. 

## Acknowledgments

The authors are grateful to Scientific Research Deanship at King Khalid University, Abha, Saudi Arabia for their financial support through the Large Research Group Projectunder grant number (RGP.02-219-43).

## Conflict of interest

The authors declare that the research was conducted in the absence of any commercial or financial relationships that could be construed as a potential conflict of interest.

## Publisher’s note

All claims expressed in this article are solely those of the authors and do not necessarily represent those of their affiliated organizations, or those of the publisher, the editors and the reviewers. Any product that may be evaluated in this article, or claim that may be made by its manufacturer, is not guaranteed or endorsed by the publisher.
